# Mitogen-Activated Protein Kinase Kinase 2, a Novel E2-Interacting Protein, Promotes the Growth of Classical Swine Fever Virus via Attenuation of the JAK-STAT Signaling Pathway

**DOI:** 10.1128/JVI.01407-16

**Published:** 2016-10-28

**Authors:** Jinghan Wang, Shucheng Chen, Yajin Liao, Enyu Zhang, Shuo Feng, Shaoxiong Yu, Lian-Feng Li, Wen-Rui He, Yongfeng Li, Yuzi Luo, Yuan Sun, Mo Zhou, Xiao Wang, Muhammad Munir, Su Li, Hua-Ji Qiu

**Affiliations:** a State Key Laboratory of Veterinary Biotechnology, Harbin Veterinary Research Institute, Chinese Academy of Agricultural Sciences, Harbin, China; b The Pirbright Institute, Pirbright, Woking, United Kingdom; Instituto de Biotecnologia/UNAM

## Abstract

The mitogen-activated protein kinase kinase/extracellular regulated kinase (MEK1/2/ERK1/2) cascade is involved in the replication of several members of the Flaviviridae family, including hepatitis C virus and dengue virus. The effects of the cascade on the replication of classical swine fever virus (CSFV), a fatal pestivirus of pigs, remain unknown. In this study, MEK2 was identified as a novel binding partner of the E2 protein of CSFV using yeast two-hybrid screening. The E2-MEK2 interaction was confirmed by glutathione *S*-transferase pulldown, coimmunoprecipitation, and laser confocal microscopy assays. The C termini of E2 (amino acids [aa] 890 to 1053) and MEK2 (aa 266 to 400) were mapped to be crucial for the interaction. Overexpression of MEK2 significantly promoted the replication of CSFV, whereas knockdown of MEK2 by lentivirus-mediated small hairpin RNAs dramatically inhibited CSFV replication. In addition, CSFV infection induced a biphasic activation of ERK1/2, the downstream signaling molecules of MEK2. Furthermore, the replication of CSFV was markedly inhibited in PK-15 cells treated with U0126, a specific inhibitor for MEK1/2/ERK1/2, whereas MEK2 did not affect CSFV replication after blocking the interferon-induced Janus kinase-signal transducer and activator of transcription (JAK-STAT) signaling pathway by ruxolitinib, a JAK-STAT-specific inhibitor. Taken together, our results indicate that MEK2 positively regulates the replication of CSFV through inhibiting the JAK-STAT signaling pathway.

**IMPORTANCE** Mitogen-activated protein kinase kinase 2 (MEK2) is a kinase that operates immediately upstream of extracellular regulated kinase 1/2 (ERK1/2) and links to Raf and ERK via phosphorylation. Currently, little is known about the role of MEK2 in the replication of classical swine fever virus (CSFV), a devastating porcine pestivirus. Here, we investigated the roles of MEK2 and the MEK2/ERK1/2 cascade in the growth of CSFV for the first time. We show that MEK2 positively regulates CSFV replication. Notably, we demonstrate that MEK2 promotes CSFV replication through inhibiting the interferon-induced JAK-STAT signaling pathway, a key antiviral pathway involved in innate immunity. Our work reveals a novel role of MEK2 in CSFV infection and sheds light on the molecular basis by which pestiviruses interact with the host cell.

## INTRODUCTION

Classical swine fever (CSF) is an economically important viral disease of pigs in many countries. The etiological agent, CSF virus (CSFV), belongs to the genus Pestivirus of the family Flaviviridae (1). The 12.3-kb genome of CSFV carries a large open reading frame that is translated into a precursor polyprotein, which is cleaved into 12 proteins, N^pro^, C, E^rns^, E1, E2, p7, NS2, NS3, NS4A, NS4B, NS5A, and NS5B (2, 3).

The E2 protein is a major envelope glycoprotein of CSFV and forms homodimers and heterodimers with E1 in virus-infected cells (4–6). The E2 protein contains four antigenic domains that are in the order B-C-D-A. Domains B and C and domains D and A each represent a globular part with a panhandle structure link in between that is anchored by a putative disulfide bond (7). Several studies have indicated that E2 is involved in virus attachment and entry (8, 9). In addition, E2 is a major determinant for virus virulence and host tropism (10). In fact, several E2-interacting host cellular proteins, including β-actin (11), annexin 2 (12), and thioredoxin 2 (13), have been identified to play important roles in the virus life cycle.

Mitogen-activated protein kinase kinases (MEKs), including MEK1 and MEK2, are tyrosine/threonine kinases that participate in the extracellular signaling-regulated kinase (ERK) signal transduction cascade (14). This cascade consists of three tiered serine/threonine kinases, Raf, MEKs, and ERKs, and regulates a large variety of biological processes, including cell migration, differentiation, metabolism, proliferation, and apoptosis (15). Two isoforms of ERKs, ERK1 and ERK2 (ERK1/2), are considered to be the only known downstream substrates of MEK1 and MEK2. It has been demonstrated that many DNA and RNA viruses utilize the cascade to replicate in host cells (16–21). Human immunodeficiency virus type 1 (HIV-1) can optimize the host cell environment for viral replication via the MEK2/ERK1/2 pathway (22). Kaposi's sarcoma-associated herpesvirus replication is modulated by the MEK1/2/ERK1/2 pathway (23, 24). Hepatitis C virus (HCV) activates MEK1/2 and ERK1/2, which enhances viral replication through attenuation of the alpha interferon (IFN-α)-induced Janus kinase-signal transducer and activator of transcription (JAK-STAT) pathway (25, 26). In addition, vesicular stomatitis virus (VSV) negatively regulates the IFN-α-induced antiviral responses through activating the cascade (27). Another study has shown that MEK2, but not MEK1, is sufficient to regulate the induction of interleukin-1 receptor antagonist (IL-1Ra) in IFN-β-activated human monocytes (28).

To date, the involvement of the MEK2/ERK1/2 signal transduction cascade in the replication of CSFV remains unknown. In the present study, we demonstrated that the CSFV E2 protein interacts with MEK2 and activates the MEK2/ERK1/2 signal transduction cascade, which in turn promotes viral replication via attenuation of the JAK-STAT signaling pathway.

## MATERIALS AND METHODS

### Cells, viruses, and plasmids.

HEK293T cells or PK-15 cells (porcine kidney cells) were grown in Dulbecco's modified Eagle's medium (DMEM) (catalog no. C11995500BT; Gibco) containing 10% fetal bovine serum (FBS) (catalog no. 12007C; Sigma-Aldrich) and maintained at 37°C in 5% CO_2_. The CSFV Shimen strain was propagated in PK-15 cells as described previously (13) and titrated using the Reed-Muench formula (29).

The bait construct pGBKT7-E2 (BD-E2) harboring the E2 gene without the transmembrane domain was generated from the CSFV Shimen strain by PCR and cloned into pGBKT7 (BD) or pGEX-6P-1. The E2 gene with the signal peptide sequence in the 5′ terminus and the Flag tag in the 3′ terminus was obtained by PCR and cloned into the pCAGGS vector (Addgene), giving rise to pCAGGS-E2-Flag. To construct the MEK2 expression vector, total cellular RNA was extracted from PK-15 cells using an RNeasy Plus minikit (catalog no. 74134; Qiagen). The gene encoding MEK2 (accession no. NM_001244550.1) was amplified by PCR and ligated into the pCMV-Myc vector (Clontech), creating pMyc-MEK2. The primers used in this study are shown in Table 1.

**TABLE 1:** Primers used in this study

Primer	Sequence (5′→3′)	Usage
BD-E2-F	CGGAATTCCGGCTAGCCTGCAAGGAAG	Amplification of E2
BD-E2-R	CTGCAGGTCGAGTCGACTCACCAGTACTG	
Flag-E2-F	CGGAATTCGCCACCATGGTATTAAGAGGACAGATCGTGC	Amplification of E2
Flag-E2-R	CATCTCGAGCTACTTGTCGTCATCGTCTTTGTAGTCTTCTGCGAAGTAA	
Flag-E2-F(690)	CGGAATTCGCCACCATGTCCGTGACATTCGAGCTC	Amplification of E2(690–823)
Flag-E2-R(823)	ATCTCGAGTTACTTGTCGTCATCGTCTTTGTAGTCTAGATCTTCATTTTCCAC	
Flag-E2-F(789)	CGGAATTCGCCACCATGGTATTAAGAGGACAGATCGTGC	Amplification of E2(789–889)
Flag-E2-R(889)	ATCTCGAGTTACTTGTCGTCATCGTCTTTGTAGTCGACAACAGGACTCGTATC	
Flag-E2-F(890)	CGGAATTCGCCACCATGTTCTACTGTAAGTTGGG	Amplification of E2(890–1053)
Flag-E2-R(1053)	ATCTCGAGTTACTTGTCGTCATCGTCTTTGTAGTCTTCTGCGAAGTAATCTGAG	
AD-MEK2-F	GAAGGATCCGCATGCTGGCCCGGAGGAAG	Amplification of MEK2
AD-MEK2-R	AGACTCGAGTCACACGGCGGTGCGC	
Myc-MEK2-F	GAAAGATCTGCATGCTGGCCCGGAGGAAG	Amplification of MEK2
Myc-MEK2-R	AGACTCGAGTCACACGGCGGTGCGC	
GST-MEK2-F(1)	GACGGATCCATGCTGGCCCGGAGGAAGCCGGTGC	Amplification of MEK2(1–265)
GST-MEK2-R(265)	AGCCTCGAGTCAGTACCTTCCGATGGACAGCTCCACC	
GST-MEK2-F(266)	GACGGATCCATGCCCATCCCCCCACCGGATGCCAAG	Amplification of MEK2(266–400)
GST-MEK2-R(400)	AGCCTCGAGTCACACGGCGGTGCGC	
FUGW-MEK2-F	ACAGGCCATTACGGCCATGCTGGCCCGGAG	Amplification of MEK2
FUGW-MEK2-R	TACGGCCGAGGCGGCCTCACACGGCGGTGCGC	

### Yeast two-hybrid screening.

The BD-E2 construct was used as bait to hybridize with a porcine primary macrophage cDNA library (13). Transformants were screened on the plates containing synthetically defined medium lacking Leu, Trp, His, and Ade (SD/−4) for 4 to 6 days. Positive colonies were further identified on SD/−4 medium containing 5-bromo-4-chloro-3-indolyl-α-d-galactopyranoside (X-α-Gal) and aureobasidin A (Aba) (SD/−4/X/Aba). The positive colonies were cultured in the SD/−4 medium and verified by sequencing as described previously (13). To validate the interaction between E2 and MEK2, the Y2HGold yeast strain was cotransformed with the plasmids BD-E2 and pGADT7-MEK2 (AD-MEK2) using yeast transformation system 2 (catalog no. 630439; Clontech). The transformants were screened on plates containing synthetically defined medium lacking Leu and Trp (SD/−2), SD/−4, or SD/−4/X/Aba. Cotransformation with pGBKT7-p53 (BD-p53)/pGADT7-T (AD-T) (coding for simian virus 40 [SV40] large T antigen), pGBKT7-Lamin (BD-Lamin) (encoding human lamin C protein)/AD-T, and BD/pGADT7 (AD) served as positive, negative, and blank controls, respectively.

### Glutathione *S*-transferase (GST) pulldown assay.

GST-tagged MEK2 mutants were expressed in Escherichia coli BL21(DE3) cells and incubated with glutathione-Sepharose 4B resin (catalog no. 17-0756-01; GE Healthcare). The resin was washed five times with phosphate-buffered saline (PBS) and incubated at 4°C for 4 h with lysates of HEK293T cells transfected with 6 μg of pCAGGS-E2-Flag or plasmids encoding Flag-tagged, truncated E2 mutants. The bound proteins in the resin were washed with PBS, followed by sodium dodecyl sulfate-polyacrylamide gel electrophoresis (SDS-PAGE) and immunoblotting analysis. GST-E2 expressed in E. coli BL21(DE3) and MEK2 expressed in HEK293T cells were also included in the GST pulldown assay to further validate the interaction as described above.

### Coimmunoprecipitation (Co-IP) assay.

HEK293T cells grown in 6-well plates were cotransfected with pMyc-MEK2 (2 μg) and pCAGGS-E2-Flag (6 μg). At 48 h posttransfection (hpt), the cells were lysed with NP-40 lysis buffer (catalog no. P0013F; Beyotime) containing 1 mM phenylmethylsulfonyl fluoride (catalog no. ST506-2; Beyotime) for 1.5 h, followed by centrifugation at 13,000 × *g* for 30 min at 4°C. The lysates were precleared with protein G-agarose (catalog no. 11243233001; Roche) and incubated for 6 h with an anti-Flag M2 affinity gel (catalog no. A2220; Sigma-Aldrich). After washing five times with PBS, the immunoprecipitated proteins were detected by SDS-PAGE and immunoblotting analysis using an anti-Myc (catalog no. C3956; Sigma-Aldrich) or an anti-Flag (catalog no. F7425; Sigma-Aldrich) polyclonal antibody (PAb).

PK-15 cells were inoculated with CSFV at a multiplicity of infection (MOI) of 1. Cell lysates collected at 24, 48, and 60 h postinfection (hpi) were subjected to Co-IP assay. After being precleared with protein G-agarose, the lysates were incubated with an anti-phosphorylated MEK2 (p-MEK2) (catalog no. 9154S; Cell Signaling Technology) or an anti-MEK2 (catalog no. 9147S; Cell Signaling Technology) monoclonal antibody (MAb). The bound proteins in the agarose were subjected to immunoblotting analysis using the anti-E2 MAb WH303 (30).

### Confocal imaging.

HEK293T cells were transiently cotransfected with pCAGGS-E2-Flag (1 μg) and pMyc-MEK2 (1 μg). PK-15 cells were mock infected or infected with CSFV at an MOI of 0.1. At 36 hpt or 48 hpi, the plasmid-transfected or CSFV-infected cells were fixed with 4% paraformaldehyde and permeabilized with 0.15% Triton X-100. The cells were further incubated with a mouse anti-Flag MAb (catalog no. F1804; Sigma-Aldrich) or anti-E2 MAb HQ06 (31) for 1.5 h, followed by incubation with a rabbit anti-Myc PAb (catalog no. C3956; Sigma-Aldrich) or a rabbit anti-MEK2 PAb (catalog no. sc-525; Santa Cruz). Subsequently, the cells were incubated with fluorescein isothiocyanate (FITC)-conjugated goat anti-mouse IgG antibody (catalog no. F2012; Sigma-Aldrich) and tetramethyl rhodamine isocyanate-conjugated goat anti-rabbit IgG antibody (whole molecule) (catalog no. T6778; Sigma-Aldrich). After incubation with 4,6-diamidino-2-phenylindole (DAPI), the cells were observed using a Leica SP2 confocal system (Leica Microsystems; Germany).

### MEK2/ERK1/2 signaling cascade inhibition assay.

The MEK1/2/ERK1/2-specific inhibitor U0126 (catalog no. s1901; Beyotime) was used to inhibit the activation of MEK1/2 (32). PK-15 cells were inoculated with CSFV as described above. At −2, 0, or 2 hpi, U0126 was added to the cells cultured in serum-free DMEM at a final concentration of 30 μM. The MEK1/ERK1/2-specific inhibitor PD98059 (catalog no. s1805; Beyotime) was used as a negative control to inhibit the activation of MEK1 (32). At 72 hpi, the supernatants were collected to detect the yields of CSFV progeny virus and viral genome copy numbers, and the cells were lysed with 100 μl of ice-cold radioimmunoprecipitation assay (RIPA) lysis buffer (catalog no. P0013C; Beyotime) containing complete protease inhibitor cocktail (catalog no. 11697498001; Roche) and phosphatase inhibitor cocktail (catalog no. 04906845001; Roche) for Western blotting.

### Indirect immunofluorescence assay (IFA).

PK-15 cells grown in 96-well plates were infected with serially diluted supernatants from CSFV-infected cells. At 72 hpi, the cells were fixed with cold absolute ethanol at −20°C for 30 min, washed with PBS five times, and incubated with an anti-E2 PAb (33). After incubation with FITC-conjugated anti-pig immunoglobulin G (IgG) (catalog no. F1638; Sigma-Aldrich), the cells were analyzed for green fluorescence using a fluorescence microscope (Nikon; Japan).

### RT-qPCR.

The viral RNA in the supernatants was extracted with a viral RNA minikit (catalog no. W7091; Watson Biotechnologies). Synthesis of cDNA was performed in a 20-μl volume containing 200 ng of total RNA, 20 U of avian myeloblastosis virus (AMV) reverse transcriptase (catalog no. D2620; TaKaRa), 200 μM deoxynucleoside triphosphates (dNTPs) (catalog no. D4030A; TaKaRa), 0.4 μM random primers (catalog no. D6045; TaKaRa), 0.5 μl of RNase inhibitor (catalog no. D2313A; TaKaRa), and 4 μl of 5× AMV reverse transcriptase buffer. Quantification of CSFV genome copy numbers was performed with Premix *Ex Taq* (Probe qPCR) (catalog no. RR390A; TaKaRa) using a previously described real-time reverse transcription-PCR (RT-qPCR) assay (34).

### Generation of a stable cell line overexpressing MEK2.

The porcine MEK2 gene was cloned into the pFUGW vector (Addgene) to generate the pFUGW-MEK2 construct. HEK293T cells were cotransfected with pFUGW-MEK2 or pFUGW and the packaging plasmids pMD2.G and psPAX2 (Addgene). At 48 hpt, the supernatants of the cell culture were harvested and filtered through a 0.22-μm-pore-size membrane, and the filtrate was ultracentrifuged to concentrate the recombinant lentiviruses. PK-15 cells were transduced with the lentiviruses at an MOI of 10 transducing units (TU). The transduced cells were passaged and analyzed for enhanced green fluorescent protein (EGFP)-tagged MEK2 (EGFP-MEK2) expression using immunoblotting at 48 h posttransduction.

### Construction of a stable cell line with MEK2 knockdown.

To knock down the expression of MEK2 in PK-15 cells, lentivirus vector pLVX-shRNA2 (Clontech)-based plasmids harboring short hairpin RNAs (shRNAs) targeting MEK2 were constructed. Briefly, shMEK2-1 (GAT CCC CGG GAG CTC AAG GAC GAT GAC TTT GAA ATT CAA GAG ATT TCA AAG TCA TCG TCC TTG AGC TCT TTT TGG AAG) and shMEK2-2 (GAT CCC CGG GGA CCA GGT GTT GAA AGA ACT CGA GTT CTT TCA ACA CCT GGT CCT TTT TGG AAG), targeting MEK2, and a nontargeting shRNA (shNC) (GAT CCC CGG TTC TCC GAA CGT GTC ACG TTT CAA GAG AAC GTG ACA CGT TCG GAG AAT TTT TGG AAG), serving as a negative control, were annealed and cloned into pLVX-shRNA2. HEK293T cells were cotransfected with the resulting recombinant plasmid pLVX-shMEK2-1, pLVX-shMEK2-2, or pLVX-shNC and the packaging plasmids pMD2.G and psPAX2. Production and transduction of lentivirus particles were performed as described above.

### Kinetics of ERK1/2 activation induced by CSFV infection.

Serum-starved PK-15 cells were infected with CSFV at an MOI of 0.1 or treated with equivalent UV-inactivated CSFV. Cell lysates collected at 0.25, 0.5, 1, 3, 6, 12, 24, and 48 hpi were immunoblotted with a rabbit anti-phosphorylated ERK1/2 (anti-p-ERK1/2) PAb (catalog no. 9102S; Cell Signaling Technology).

### JAK-STAT inhibition assay.

PK-15 cells cultured in 24-well plates were treated with U0126 at a final concentration of 5 μM for 4 h, followed by washing twice with PBS. The cells were incubated with 0.25 μM ruxolitinib (Ruxo) (catalog no. 941678; Nce Biomedical) and 10 international units (IU) of porcine IFN-α (catalog no. RP0010S-005; Kingfisher) for 6 h. The cells were washed with PBS and infected with CSFV as described above. Subsequently, the cells were maintained in DMEM containing 5 μM U0126 and 10 IU of porcine IFN-α. At 72 hpi, the supernatants were collected to detect the yields of CSFV progeny virus and viral genome copy numbers, and the cell lysates were examined by Western blotting.

The MEK2-overexpressing or MEK2 knockdown cells were infected with 0.1 MOI of CSFV for 1.5 h. After washing with DMEM, the cells were treated with 10 IU of porcine IFN-α for 2 h and incubated with fresh DMEM containing 3% FBS and 0.25 μM Ruxo. At 60 hpi, the supernatants were collected for detection of viral genome copy numbers and progeny virus titers, and the cell lysates were subjected to Western blotting.

### Statistical analysis.

Statistical analyses were conducted using SPSS 17.0 software. Student's *t* test and one-way analysis of variance (ANOVA) were used to compare CSFV titers or viral genome copy numbers.

## RESULTS

### The CSFV E2 protein interacts with MEK2.

**FIG 1:**
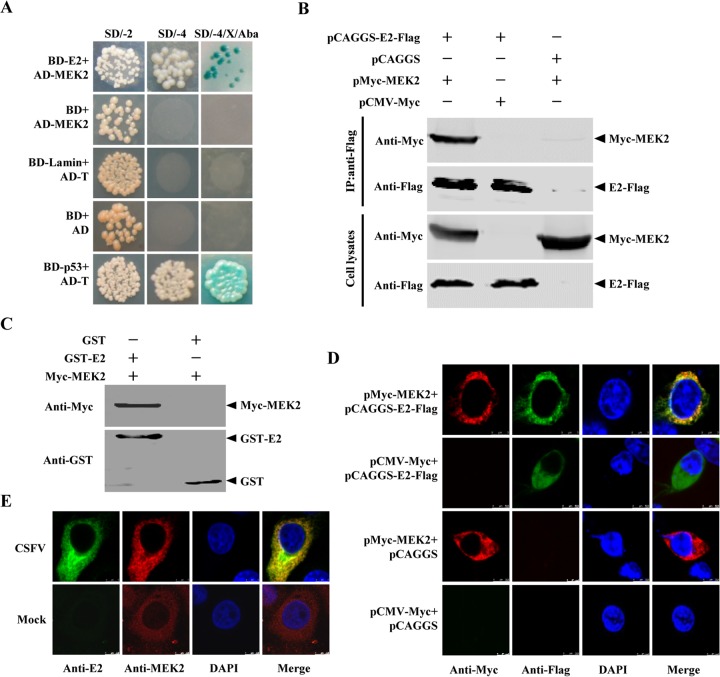
Interaction between the CSFV E2 protein and MEK2. (A) Yeast cotransformation assay. The Y2HGold yeast strain was cotransformed with pGBKT7-E2 (BD-E2)/pGADT7-MEK2 (AD-MEK2), pGBKT7-p53 (BD-p53)/pGADT7-T (AD-T) (positive control), or pGBKT7-Lamin (BD-Lamin)/AD-T (negative control). (B) Coimmunoprecipitation (Co-IP) analysis of MEK2 and E2. HEK293T cells were cotransfected with pMyc-MEK2 and pCAGGS-E2-Flag. Cells collected at 48 h posttransfection (hpt) were lysed, precleared with protein G-agarose, and incubated with anti-Flag M2 affinity gel for 6 h at 4°C. The proteins were analyzed by Western blotting using a rabbit anti-Flag or anti-Myc polyclonal antibody (1:500). (C) GST pulldown assay. GST or GST-E2 expressed in Escherichia coli BL21(DE3) was purified with a glutathione-Sepharose 4B resin (catalog no. 10049253; GE Healthcare) and incubated with Myc-MEK2 expressed in HEK293T cells. The bound proteins were subjected to Western blotting using the indicated antibodies. (D and E) Colocalization of MEK2 with E2. HEK293T cells cotransfected with pMyc-MEK2 and pCAGGS-E2-Flag (D) or PK-15 cells infected with CSFV (E) were examined by indirect immunofluorescence assay (IFA) for expression of MEK2 (red) and E2 (green).

In this study, MEK2 was selected for further study, considering the involvement of MEK2 in the MEK2/ERK1/2 signaling pathway (14). The prey plasmid screened from the positive yeast clones was found to harbor an incomplete MEK2 containing amino acids (aa) 186 to 400 of the porcine MEK2. However, this truncated MEK2 still contains the whole proline-rich domain (aa 266 to 334) and almost all phosphorylation sites of MEK2. To preclude possible self-activation, yeast cotransformation was performed, and only the BD-E2/AD-MEK2-cotransformed Y2HGold strain grew well in SD/−4/X/Aba medium (Fig. 1A). To further validate the MEK2-E2 interaction, a Co-IP assay was conducted using the lysates from HEK293T cells overexpressing Flag-tagged E2 and Myc-tagged MEK2. After incubating with anti-Flag M2 affinity gel, the Flag-tagged E2 was found to coprecipitate with the Myc-tagged MEK2 (Fig. 1B). A GST pulldown assay was conducted with the GST-tagged E2 protein and the Myc-tagged MEK2 protein. The results showed that GST-E2 but not GST interacted with MEK2 (Fig. 1C). The localization of E2 and MEK2 in the cotransfected or CSFV-infected cells was analyzed for double-label immunofluorescence using confocal microscopy. The results showed a clear colocalization of E2 and MEK2 in the cytoplasm of the cells (Fig. 1D and E). On the basis of digital analysis of the cell images, the colocalization coefficients were determined to be 0.942 and 0.835 in cotransfected and infected cells, respectively. Collectively, the data indicate that the E2 protein of CSFV interacts with MEK2.

### The MEK2-E2 interaction occurs during the late stage of CSFV replication and depends on the C termini of MEK2 and E2.

**FIG 2:**
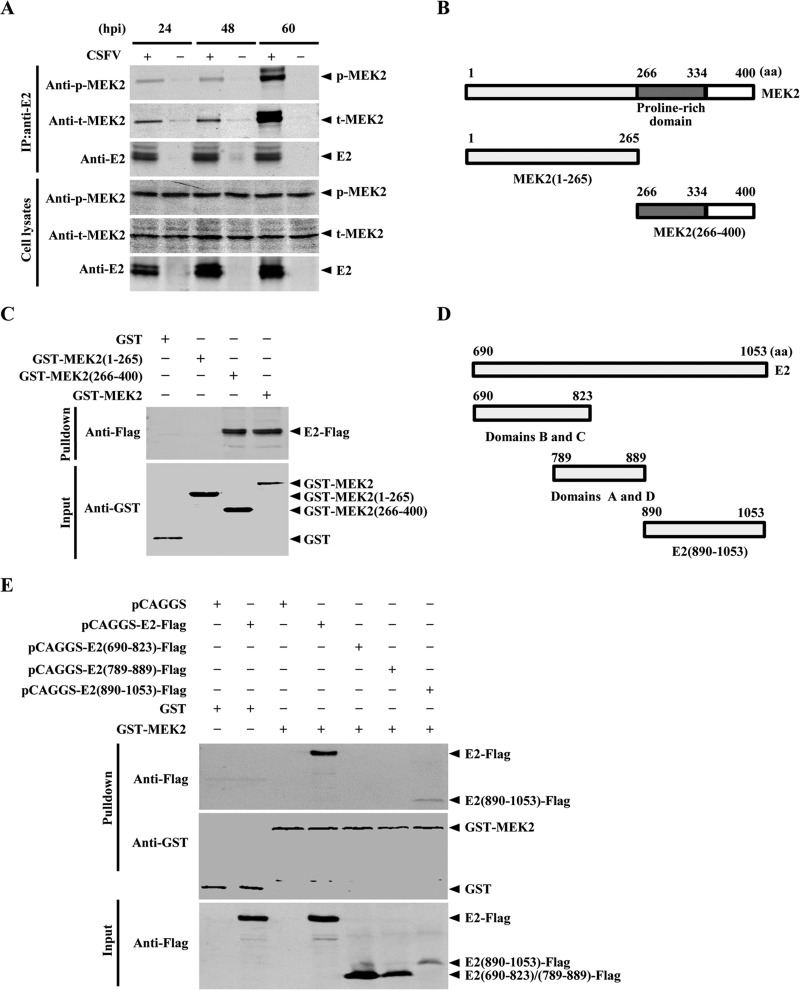
The MEK2-E2 interaction occurs during the late stage of CSFV replication and requires the C termini of MEK2 and E2. (A) Co-IP analysis of E2 and MEK2 in CSFV-infected cells. PK-15 cells were infected with CSFV, and the cells lysates were collected at 24, 48, and 60 h postinfection (hpi) and precleared with protein G-agarose, followed by incubation with a rabbit anti-phosphorylated MEK2 (anti-p-MEK2) or a rabbit anti-MEK2 monoclonal antibody (MAb) (1:500) at 4°C for 6 h and incubation with protein G-agarose at 4°C for 6 h. The bound proteins in the agarose were examined by Western blotting using anti-E2 MAb WH303 (1:200). (B) Schematic representation of the porcine MEK2 protein domains and individual MEK2 deletion mutants. (C) GST pulldown analysis of the interaction of GST-tagged MEK2 or its mutants with Flag-tagged E2 expressed in HEK293T cells. The recombinant protein of GST-MEK2 or GST-tagged MEK2 mutants was incubated with glutathione-Sepharose 4B resin (catalog no. 17-0756-01; GE Healthcare). The resin was washed with PBS and incubated with the Flag-tagged E2 protein expressed in HEK293T cells. Immunoblotting analysis was conducted to detect the bound proteins. (D) Schematic representation of the truncated E2 mutants. (E) GST pulldown analysis of GST-tagged MEK2 and Flag-tagged, truncated E2 mutants. The recombinant protein GST-MEK2 was incubated with glutathione-Sepharose 4B resin and then with a series of Flag-tagged, truncated E2 mutants. The bound proteins in the resin were then analyzed by Western blotting as described above.

To examine MEK2-E2 interaction during the replication cycle of CSFV infection and to investigate the dependency of this interaction on the activation of MEK2, we collected the lysates from CSFV-infected PK-15 cells at 24, 48, and 60 hpi. The Co-IP assay demonstrated that the MEK2-E2 interaction occurs in the late phase of CSFV replication that is independent of MEK2 activation (Fig. 2A).

To determine the critical domain of MEK2 that is essential for the interaction with E2, a series of MEK2 deletion mutants were generated based on its domains (Fig. 2B) and tested by a GST pulldown assay. The results demonstrated that the C terminus of MEK2 (aa 266 to 400) mediates the MEK2-E2 interaction (Fig. 2C).

To map the key domain(s) of E2 required for the interaction with MEK2, we constructed plasmids expressing a series of truncated E2 mutants based on the antigenic structure of the E2 protein (Fig. 2D) and examined the interaction with MEK2 using a GST pulldown assay. The results indicated that the C terminus of E2 (aa 890 to 1053) mediates the MEK2-E2 interaction (Fig. 2E).

### MEK2 enhances CSFV growth.

**FIG 3:**
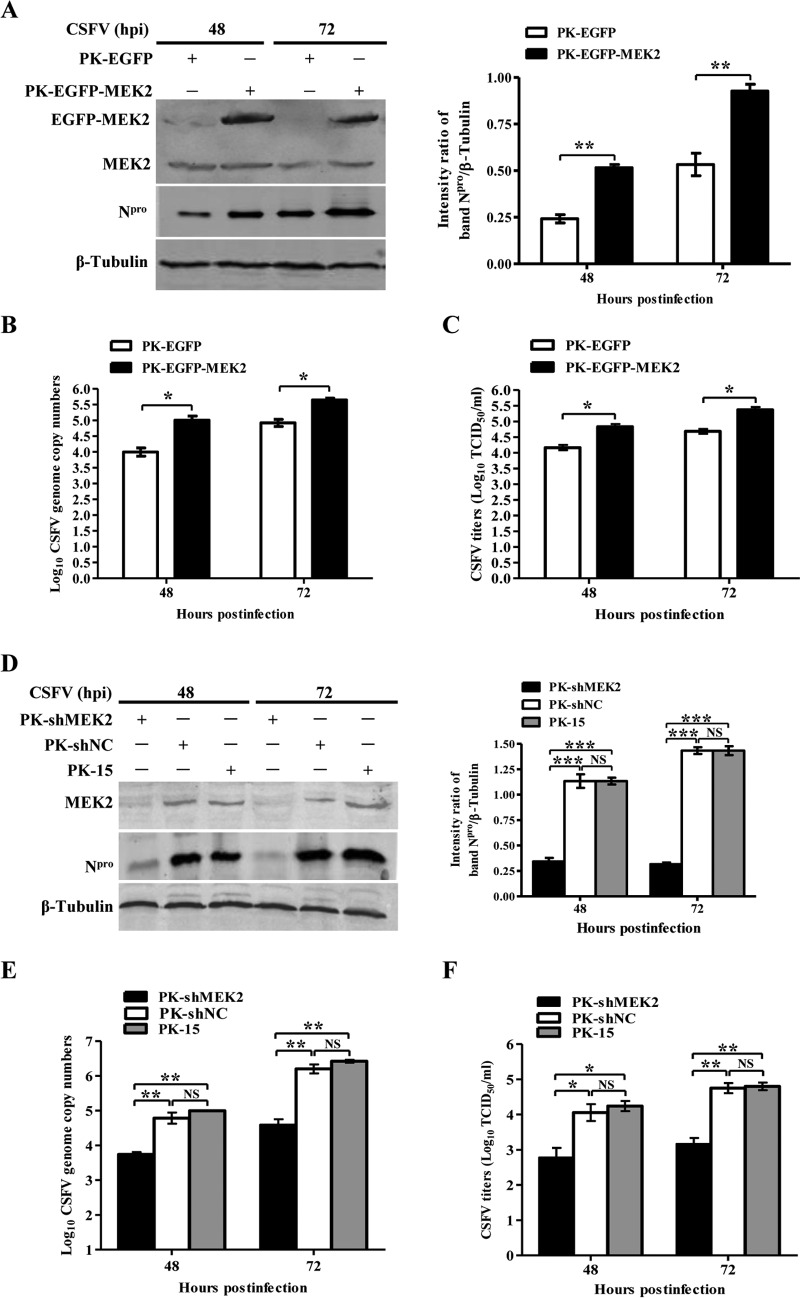
MEK2 positively modulates CSFV replication. PK-15 cells transduced with Lenti-EGFP-MEK2 or Lenti-EGFP were infected with CSFV at a multiplicity of infection (MOI) of 0.1 for 48 h or 72 h. (A) Western blotting of N^pro^ or MEK2 expression in a stable cell line overexpressing MEK2. β-Tubulin was included as an internal reference. Quantification analysis of N^pro^ protein expression was conducted using the Odyssey application software version 3.0. (B) Viral genome copy numbers of MEK2-overexpressing cells infected with CSFV. CSFV genome copy numbers in MEK2-overexpressing cells were assessed using a real-time reverse transcription-PCR assay at 48 or 72 h postinfection (hpi). (C) Infectious progeny virus titers in the supernatants of MEK2-overexpressing cells infected with CSFV. Viral titers in the supernatants were assayed at 48 or 72 hpi and expressed as 50% tissue culture infective doses (TCID_50_)/ml. PK-15 cells transduced with Lenti-shMEK2 or Lenti-shNC were inoculated with CSFV. (D) Expression of the CSFV N^pro^ protein in PK-15 cells with knockdown of MEK2 by lentivirus-mediated shRNAs. (E) CSFV genome copy numbers in MEK2 knockdown cells. (F) CSFV titers in MEK2 knockdown cells. Means ± standard deviations (SD) for three technical replicates are shown. *, *P* < 0.05; **, *P* < 0.01; ***, *P* < 0.001; NS, not significant (*P* > 0.05).

To clarify the effects of MEK2 on CSFV replication, we examined the virus growth in MEK2-overexpressing (PK-EGFP-MEK2) or MEK2 knockdown (PK-shMEK2) cells. The results showed that overexpression of MEK2 increased the expression of the N^pro^ protein in PK-EGFP-MEK2 cells (Fig. 3A). The viral genome copy numbers and viral titers of the supernatants were augmented significantly (Fig. 3B and C). In contrast, knockdown of MEK2 resulted in the reduction of N^pro^ protein expression compared to that in the control cells (Fig. 3D). The viral genome copy numbers and titers were also decreased in the supernatants of CSFV-infected cells (Fig. 3E and F). Collectively, the gain-of-function and complementary loss-of-function experiments confirm that MEK2 positively regulates CSFV replication.

### CSFV induces a biphasic activation of ERK1/2.

**FIG 4:**
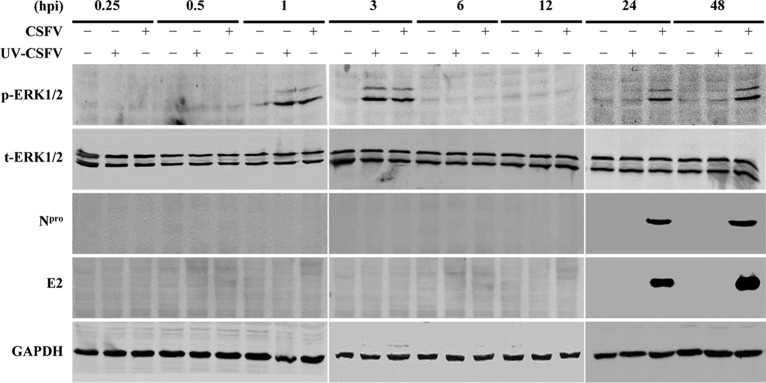
CSFV infection results in a biphasic activation of ERK1/2. PK-15 cells were left uninfected, infected with CSFV at a multiplicity of infection (MOI) of 0.1, or treated with an equal amount of UV-inactivated CSFV (UV-CSFV). Cell lysates collected at 0.25, 0.5, 1, 3, 6, 12, 24, and 48 hpi were used for Western blotting to analyze the expression of phosphorylated ERK1/2 (p-ERK1/2), total ERK1/2 (t-ERK1/2), N^pro^, and E2.

To determine the kinetics of ERK1/2 activation induced by CSFV infection, serum-starved PK-15 cells were infected with live or UV-inactivated CSFV. The results showed that CSFV infection of PK-15 cells induced ERK1/2 phosphorylation with a first transient peak at 3 hpi and a second peak at 24 hpi. However, the inactivated CSFV was sufficient to trigger ERK1/2 activation at the early but not the late stage of viral infection (Fig. 4), indicating that a biphasic activation of ERK1/2 is induced by CSFV infection.

### Inhibition of the MEK2/ERK1/2 cascade suppresses the replication of CSFV.

**FIG 5:**
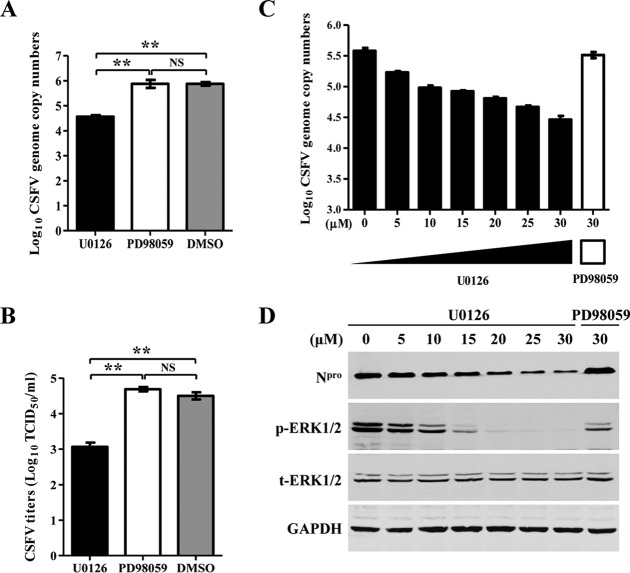
Inhibition of the MEK2/ERK1/2 cascade suppresses the replication of CSFV. (A and B) U0126 but not PD98059 inhibits CSFV replication. PK-15 cells were inoculated with CSFV for 1.5 h. After washing twice with DMEM, the cells were further cultured for 72 h in the presence of U0126 (catalog no. s1901; Beyotime), PD98059 (catalog no. s1805; Beyotime), or DMSO (catalog no. D2650; Sigma-Aldrich). The supernatants were collected at 72 hpi for determining the viral genome copy numbers (A) and viral titers (B). (C and D) Blockage of the MEK2/ERK1/2 cascade inhibits CSFV replication in a dose-dependent manner. To further determine whether the MEK2/ERK1/2 cascade suppresses CSFV growth, U0126 was added to the medium at a concentration of 0, 5, 10, 15, 20, 25, or 30 μM, and 30 μM PD98059 was used as a negative control. At 72 h postinfection (hpi), the supernatants were collected. Viral genome copy numbers were determined (C), and the cell lysates were subjected to Western blotting to determine the expression of N^pro^, phosphorylated ERK1/2 (p-ERK1/2), and total ERK1/2 (t-ERK1/2) (D). Means ± standard deviations (SD) for three technical replicates are shown. *, *P* < 0.05; **, *P* < 0.01; ***, *P* < 0.001; NS, not significant (*P* > 0.05).

To further confirm that MEK2 participates in the regulation of CSFV replication, U0126 or PD98059 (control) was added to the medium of CSFV-infected PK-15 cells. Both inhibitors at up to 30 μM did not affect the cell viability (data not shown), and U0126 but not PD98059 decreased viral genome copy numbers (Fig. 5A) and viral titers (Fig. 5B) in the supernatants of the CSFV-infected cells.

After blocking the cascade with various concentrations (0 to 30 μM) of the inhibitor U0126 in CSFV-infected PK-15 cells, the viral genome copy numbers in the supernatants and the N^pro^ expression in the cell lysates were decreased in a dose-dependent manner (Fig. 5C and D). The results indicate that inhibition of the MEK2/ERK1/2 cascade suppresses CSFV replication.

### Inhibition of MEK2/ERK1/2 activation affects the postattachment step of the CSFV life cycle.

**FIG 6:**
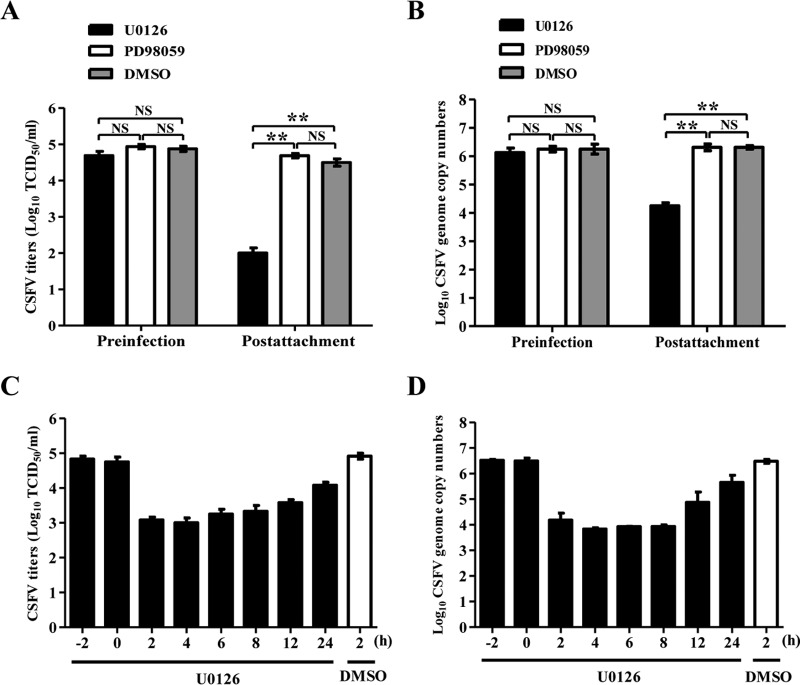
U0126 does not affect CSFV attachment. (A and B) U0126 inhibits CSFV replication in the postattachment stage. For the preinfection group, the MEK2-specific inhibitor U0126 (15 μM) was added to the medium at 2 h preinfection with CSFV. At 1.5 h postattachment, the cells were washed five times with phosphate-buffered saline (PBS), and the medium was replaced with fresh medium without U0126. For the postattachment group, the inhibitor U0126 was added to the medium at 2 h postattachment and kept in the medium throughout the experiment. The supernatants were collected, and viral titers (A) and viral genome copy numbers (B) were determined at 72 postinfection (hpi). (C and D) U0126 inhibits CSFV replication. At 1.5 h postattachment, the cells were washed with PBS and incubated with fresh medium at 1.5 hpi. The inhibitor U0126 (15 μM) was added to the medium at various time points (2, 4, 6, 8, 10, 12, or 24 hpi) and kept in the medium throughout the experiment, except for the preinfection group (−2 or 0 hpi). The supernatants were collected at 72 hpi, and virus titers (C) and viral genome copy numbers (D) were determined. Means ± standard deviations (SD) for three technical replicates are shown. *, *P* < 0.05; **, *P* < 0.01; ***, *P* < 0.001; NS, not significant (*P* > 0.05).

To investigate the specific stage of CSFV infection that was targeted by the inhibition of ERK1/2 activation, the inhibitor U0126 was added to the medium at different time points, and then the CSFV-infected PK-15 cells were cultured until 72 hpi. In comparison with the dimethyl sulfoxide (DMSO)-treated control, U0126 treatment prior to virus infection did not influence CSFV growth (Fig. 6A and B). In contrast, the inhibitor significantly suppressed the replication of CSFV in PK-15 cells treated with U0126 after virus attachment (Fig. 6C and D).

### The MEK2/ERK1/2 cascade enhances CSFV replication through the JAK-STAT signaling pathway.

**FIG 7:**
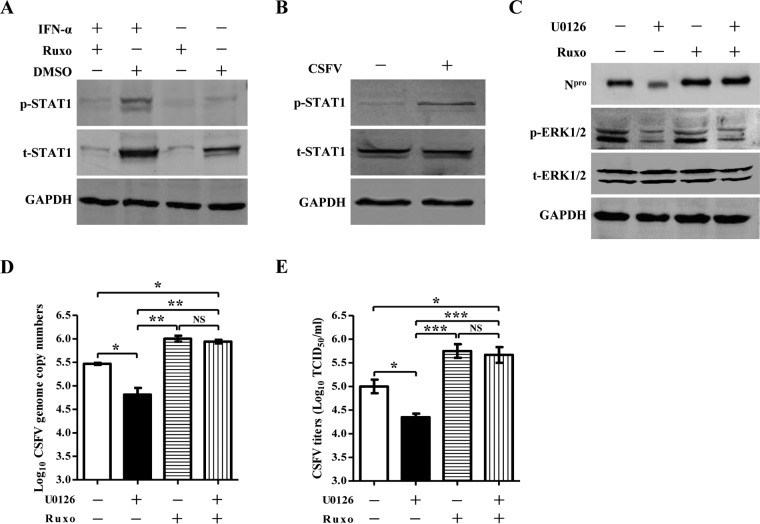
Inhibition of the interferon-induced JAK-STAT signaling pathway abolishes the increase of CSFV replication by the MEK2/ERK1/2 cascade. (A) Blockage of the JAK-STAT pathway with ruxolitinib (Ruxo). PK-15 cells were treated with Ruxo (catalog no. 941678; Nce Biomedical) or DMSO (catalog no. D2650; Sigma-Aldrich), and phosphorylated STAT1 (p-STAT1) was tested to confirm the inhibition of the JAK-STAT signaling pathway by Ruxo. The expression of total STAT1 (t-STAT1) and glyceraldehyde-3-phosphate dehydrogenase (GAPDH) was also examined. (B) CSFV infection induces expression of p-STAT1. PK-15 cells were infected with CSFV at a multiplicity of infection (MOI) of 0.1. At 1 h postinfection, the expression of p-STAT1 was detected by Western blotting using an anti-p-STAT1 (Tyr701) monoclonal antibody. (C) Viral protein expression in the U0126- and Ruxo-treated cells. PK-15 cells were incubated with U0126 (catalog no. s1901; Beyotime) and subsequently treated with Ruxo and IFN-α (catalog no. RP0010S-005; Kingfisher), followed by CSFV infection. After removal of the virus inocula, the cells were maintained in DMEM containing U0126 and IFN-α. The CSFV N^pro^ protein expression at 60 h postinfection (hpi) was checked by immunoblotting analysis. (D and E) Detection of CSFV genome copy numbers (D) and viral titers (E) in U0126- and Ruxo-treated cells. Means ± standard deviations (SD) for three technical replicates are shown. *, *P* < 0.05; **, *P* < 0.01; ***, *P* < 0.001; NS, not significant (*P* > 0.05).

To investigate whether the MEK2/ERK1/2 cascade enhances CSFV replication via interference with the JAK-STAT pathway, we utilized Ruxo, a JAK-STAT-specific inhibitor, to block the pathway and examined the effects of Ruxo on the increase of CSFV growth by the MEK2/ERK1/2 pathway. Blocking the pathway with Ruxo resulted in reduced expression of phosphorylated STAT1 (p-STAT1) induced by IFN-α (Fig. 7A). Expression of p-STAT1 in PK-15 cells was detected during the early phase of CSFV infection (Fig. 7B). After blocking the pathway, U0126 did not affect the viral protein expression (Fig. 7C), genome copy numbers (Fig. 7D), and titers (Fig. 7E), indicating that activation of the MEK2/ERK1/2 cascade enhances CSFV replication via attenuation of the IFN-induced JAK-STAT signaling pathway.

### MEK2 cannot promote CSFV replication following blockade of the JAK-STAT signaling pathway.

**FIG 8:**
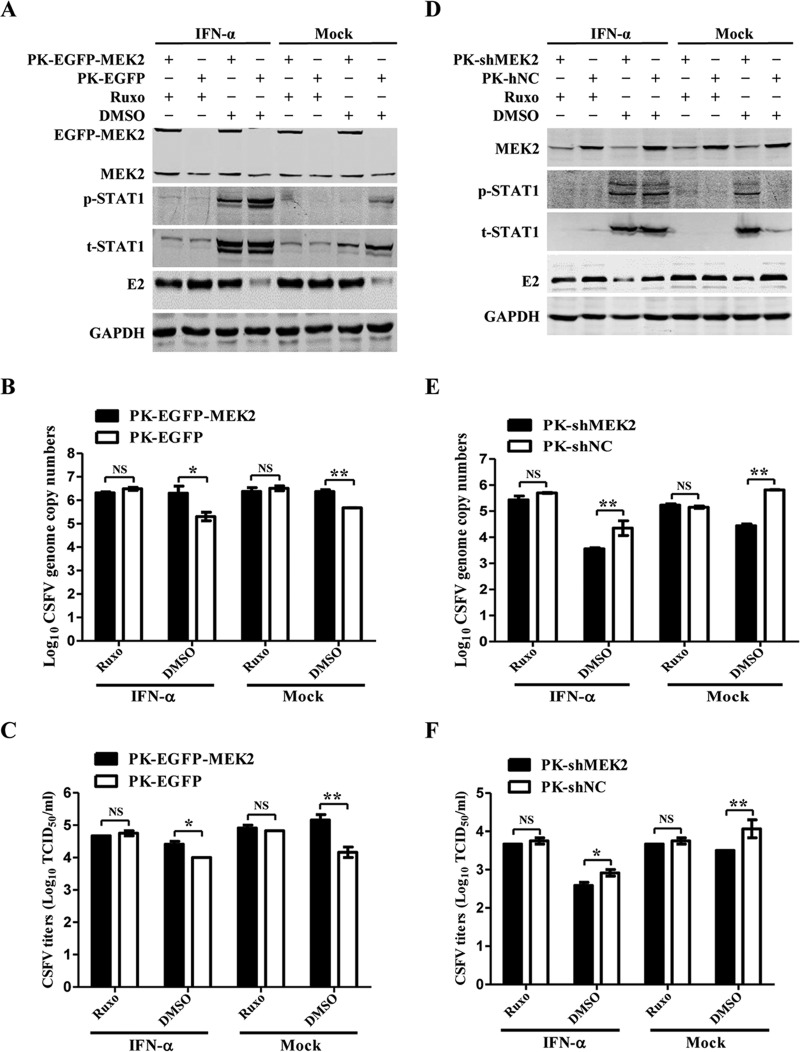
MEK2 cannot promote CSFV replication after blockage of the JAK-STAT signaling pathway. (A to C) CSFV replication in IFN-α- and Ruxo-treated MEK2-overexpressing PK-15 cells. PK-EGFP-MEK2 or PK-EGFP cells were treated with IFN-α and Ruxo, followed by CSFV infection. After removal of the virus inocula, the cells were cultured with DMEM containing IFN-α and ruxolitinib (Ruxo). The CSFV N^pro^ protein expression at 60 h postinfection (hpi) was determined by immunoblotting analysis (A), CSFV genome copy numbers by real-time reverse transcription-PCR (B), and viral titers by titration (C) for the IFN-α- and Ruxo-treated PK-EGFP-MEK2 cells. (D to F) CSFV replication in IFN-α- and Ruxo-treated MEK2 knockdown cells. The CSFV N^pro^ protein expression (D), CSFV genome copy numbers (E), and viral titers (F) in the IFN-α- and Ruxo-treated PK-shMEK2 or PK-shNC cells were examined as described above. Means ± standard deviations (SD) for three technical replicates are shown. *, *P* < 0.05; **, *P* < 0.01; ***, *P* < 0.001; NS, not significant (*P* > 0.05).

To further define whether MEK2 enhances CSFV replication via inhibition of the JAK-STAT signaling pathway, we blocked the cascade using Ruxo and examined the effects of MEK2 on CSFV replication. After blocking the pathway, overexpression of MEK2 failed to promote viral protein expression (Fig. 8A), viral genome copy numbers (Fig. 8B), and viral titers (Fig. 8C), while knockdown of MEK2 was unable to inhibit CSFV growth (Fig. 8D to F), indicating that MEK2 enhances CSFV replication via attenuation of the JAK-STAT signaling pathway.

## DISCUSSION

Viruses have evolved to regulate the signaling pathways of the host cell for viral replication. Here, for the first time, we demonstrate that the MEK2/ERK1/2 cascade is required for efficient replication of CSFV in cultured cells. The MEK1/2/ERK1/2 cascade is also involved in viral infection by several Flaviviridae members. It has been reported that this cascade promotes HCV replication (26, 35). In addition, West Nile virus (WNV) induces a transient activation of ERK1/2 at the early stage of infection (36). Furthermore, inhibition of ERK1/2 phosphorylation by U0126 results in a dose-dependent decrease of dengue virus 2 replication (37). Notably, we demonstrate that inhibition of the MEK2/ERK1/2 cascade activation by U0126 severely impairs virus production in PK-15 cells during the postattachment step of the CSFV life cycle, indicating the potential of U0126-derived agents as antivirals against CSFV infection.

In this study, we demonstrate that CSFV infection triggers a biphasic activation of ERK1/2, indicating that the two phases result from different mechanisms. The first phase of ERK1/2 activation is likely attributable to CSFV-receptor interaction and internalization, whereas the second phase of activation possibly is due to viral protein synthesis. In addition, the biphasic activation of ERK1/2 has also been observed for coxsackievirus B3 (38), enterovirus 71 (EV71) (18), and herpes simplex virus 2 (HSV-2) (39). The UV-inactivated virus retains the ability for receptor binding and endocytosis into host cells but fails to replicate the viral genome and express viral proteins. PK-15 cells exposed to the inactivated CSFV efficiently induced ERK1/2 activation at the early stage of infection, indicating that virus entry is essential for the early ERK1/2 activation. The receptor-mediated signaling cascade is an initial event where cells recognize and bind to the ligands, followed by delivery of the extracellular signal to the intracellular signaling networks (25). The HCV E2 protein has been shown to activate the MEK1/2/ERK1/2 cascade, whereas the activation of E2-induced MEK1/2/ERK1/2 is decreased upon blockage of CD81 or low-density lipoprotein receptor using corresponding antibodies (40). Whether the CSFV E2 protein binds to the cells and subsequently activates the cascade requires further study. The present study showed that MEK2 interacts with the CSFV E2 protein and positively regulates CSFV replication, indicating that MEK2 possibly functions as a link between CSFV production and the MEK2/ERK1/2 cascade.

Increasing numbers of studies have shown that MEK1 and MEK2 have distinct functions in a wide variety of cells (41–44). Inhibition of ERK1/2 activation by U0126 or knockdown of MEK1 by small interfering RNA (siRNA) remarkably impaired HSV-2 production, whereas silencing of MEK2 had little effect (21). The same phenomenon has also been observed in EV71 infection (18). We showed here that U0126 but not PD98059 inhibits CSFV replication. The distinct effects of U0126 and PD98059 on the replication of CSFV suggest that the inhibitor of MEK2 displays specific inhibition of CSFV growth. In addition, by using overexpression or knockdown of MEK2 mediated by lentivirus, we demonstrated that MEK2 can significantly upregulate the CSFV replication. These findings not only provide new insight into understanding CSFV utilization of the cellular MEK2/ERK1/2 signaling pathway to infect the host cell but also indicate a potential antiviral therapy for CSFV.

The cellular activity of the Raf/MEK1/2/ERK1/2 pathway may influence cellular susceptibility to IFN, which in turn affects host antagonism against the virus. Previous studies have shown that activation of Raf/MEK1/2/ERK1/2 negatively regulates the IFN-α-induced antiviral response (27, 45). However, the cross-linking function between the Raf/MEK1/2/ERK1/2 and JAK-STAT pathways has not been illustrated in different cell lines. We have previously shown that addition of recombinant IFN-β to CSFV-infected cells dramatically decreases the yields of progeny virus (46). In the present work, we show that the expression of p-STAT1 in PK-15 cells is induced during the early phase of CSFV infection, indicating that CSFV can activate the JAK-STAT pathway. Furthermore, we demonstrate that MEK2 no longer influences CSFV replication after blockage of the JAK-STAT signaling pathway with Ruxo and that knockdown of MEK2 promotes the expression of p-STAT1, indicating that MEK2 enhances CSFV replication via attenuation of the pathway. This has also been observed in cells infected with HCV (25). The precise mechanism by which MEK2 negatively modulates the expression of the STAT1 and the JAK-STAT signaling pathway in PK-15 cells needs further investigation.

In conclusion, we show that cellular MEK2 acts as a novel interacting partner of the CSFV E2 protein and enhances CSFV replication via attenuation of the JAK-STAT signaling pathway. Further studies are needed to elucidate the exact molecular mechanisms by which MEK2 or other components of the cascade negatively modulate the JAK-STAT pathway in CSFV-permissive cells.
